# High dose intravenous vitamin C treatment in Sepsis: associations with acute kidney injury and mortality

**DOI:** 10.1186/s12882-021-02599-1

**Published:** 2021-11-20

**Authors:** Thomas R. McCune, Angela J. Toepp, Brynn E. Sheehan, Muhammad Shaheer K. Sherani, Stephen T. Petr, Sunita Dodani

**Affiliations:** 1 Division of Nephrology, Department of Internal Medicine, Eastern Virginia Medical School, 301 Riverview Ave, Suite 600, Norfolk, Virginia 23510 USA; 2 EVMS-Sentara Healthcare Analytics and Delivery Science Institute, Eastern Virginia Medical School, Norfolk, Virginia USA; 3 Department of Internal Medicine, Eastern Virginia Medical School, Norfolk, Virginia USA; 4 Department of Psychiatry and Behavioral Sciences, Eastern Virginia Medical School, Norfolk, Virginia USA; 5 Eastern Virginia Medical School, Norfolk, Virginia USA; 6 Division of Cardiology, Department of Internal Medicine, Eastern Virginia Medical School, Norfolk, Virginia USA

**Keywords:** Vitamin C therapy, Sepsis, Acute kidney injury, In-hospital mortality

## Abstract

**Background:**

The effects of vitamin C on clinical outcomes in critically ill patients remain controversial due to inconclusive studies. This retrospective observational cohort study evaluated the effects of vitamin C therapy on acute kidney injury (AKI) and mortality among septic patients.

**Methods:**

Electronic medical records of 1390 patients from an academic hospital who were categorized as Treatment (received at least one dose of 1.5 g IV vitamin C, *n* = 212) or Comparison (received no, or less than 1.5 g IV vitamin C, *n* = 1178) were reviewed. Propensity score matching was conducted to balance a number of covariates between groups. Multivariate logistic regressions were conducted predicting AKI and in-hospital mortality among the full sample and a sub-sample of patients seen in the ICU.

**Results:**

Data revealed that vitamin C therapy was associated with increases in AKI (OR = 2.07 95% CI [1.46–2.93]) and in-hospital mortality (OR = 1.67 95% CI [1.003–2.78]) after adjusting for demographic and clinical covariates. When stratified to examine ICU patients, vitamin C therapy remained a significant risk factor of AKI (OR = 1.61 95% CI [1.09–2.39]) and provided no protective benefit against mortality (OR = 0.79 95% CI [0.48–1.31]).

**Conclusion:**

Ongoing use of high dose vitamin C in sepsis should be appraised due to observed associations with AKI and death.

## Clinical summary

- Existing research regarding the effect of vitamin C on outcomes in patients with sepsis is inconsistent.
- This retrospective study of patients hospitalized for sepsis revealed an association between IV vitamin C usage and subsequent AKI and in-hospital mortality.
- The relationship between early treatment of high dose IV vitamin C and mortality in septic patients might suggest potential nephrotoxicity of vitamin C therapy.
- Findings, coupled with results from multiple randomized controlled prospective studies of IV vitamin C with mixed results, suggests that wide-spread use of high dose IV vitamin C requires further assessment.

## Background

The benefits of ascorbic acid (vitamin C) therapy were first noted in the 1970s, related to infections and the common cold [1]. The unique set of physiological properties of vitamin C have given its use promise in decelerating a multitude of different forms of infection. Vitamin C serves to protect host cells from oxidative damage during the inflammatory response through its role as an antioxidant [2], it preserves other endogenous antioxidants, including lipid soluble vitamin E and glutathione [3], and it has been shown to contribute to bactericidal activity via augmentation of T-cell and neutrophil function [4]. As plasma vitamin C is quickly depleted during severe inflammatory states, vitamin C has emerged as a possible adjuvant therapy in sepsis [5, 6].

Sepsis remains a major public health issue as the 10th leading cause of death among non-Hispanic black individuals and 12th among non-Hispanic white individuals in the U.S., and infecting more than 1.7 million U.S. Americans annually [7, 8]. Within the hospital setting, sepsis remains a significant concern with mortality rates above 50% and over $24 billion dollars spent each year on sepsis-related hospitalizations [9–13]. Identifying effective and appropriate treatments to reduce sepsis-related mortality and hospital length of stay (LOS) remains a challenge.

Early examinations of vitamin C supplementation, alone or in combination with thiamine (vitamin B1) and corticosteroids, revealed decreased inflammatory markers and end-organ dysfunction [14, 15]. Extant research, however, is inconsistent regarding the effect of vitamin C on outcomes in patients with sepsis. Of six randomized controlled trials, only two found improvement in patient markers such as SOFA score, vasopressor requirement, and lactate levels [14, 16–20]. Only one study found reduced likelihood of mortality with vitamin C therapy; however, notably, this study did not find improvement in SOFA score [17]. Observational studies also remain mixed. One of three retrospective and quasi-experimental studies found improvements in both mortality and markers of patient stability, a second found a reduction in mortality but no benefit for markers of stability, and a third found no benefit of mortality or markers of stability [15, 21, 22].

Similarly, research assessing the role of high dose vitamin C in causing or protected against acute kidney injury (AKI) is also conflicting and lacks validity. Several case reports have identified AKI and oxalate nephropathy in patients who received high doses of vitamin C [23–25]. Other research has suggested vitamin C may have some level of protectiveness against AKI [15] while other research including the large clinical trial, VICTAS, found there was no harmful or beneficial relationship [26, 27]. Given the contradictory findings in both RCTs and observational studies, more research is needed to ascertain the effects of vitamin C therapy among septic patients. In the current study, a retrospective analysis was conducted to investigate the relationship of intravenous (IV) vitamin C on AKI and mortality among patients with sepsis.

## Methods

### Clinical order set

In August 2016, a sepsis vitamin order set was formalized but not mandated in the hospital in which the study was completed. The decision to utilize the order set was up to the individual provider. The order panel included IV vitamin C at a dose of 1.5 g, administered every 6 h for a maximum of 16 doses; IV hydrocortisone 50 mg every 6 h up to 28 doses; and IV thiamine 200 mg every 12 h for 8 doses. In December 2018, melatonin 0.5 mg at bedtime was added to the order set with no stop required. The order panel did not include diagnostic studies to determine the severity of sepsis nor did it include treatment option directions based on clinical parameters.

### Study design

**Fig. 1:**
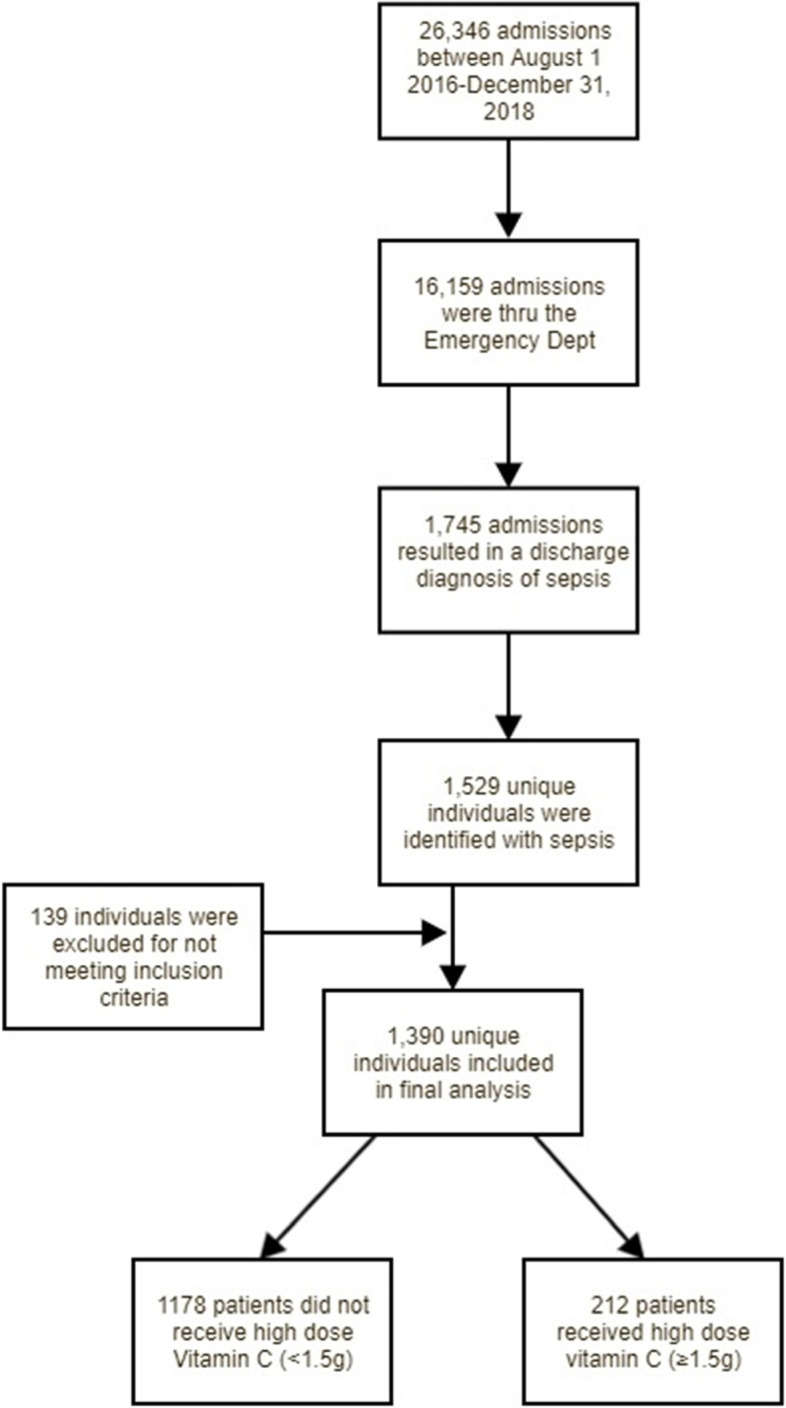
Study Design

This retrospective cohort study (Fig. 1) was conducted at a tertiary care, academic hospital and was approved by the Eastern Virginia Medical School Institutional Review Board. Adult patients, age 18 through 89 years, with International Classification of Disease (ICD)-10 code for “sepsis”, admitted between August 1, 2016 and December 31, 2018, were identified via electronic health records (EHR-Epic Systems Corporation, Verona, WI, USA). Inclusion criteria included (1) admission via the ED, (2) hospitalization for at least 48 h, and (3) two serum creatinine assays 48 h apart. ED admission was required in order to capture patients with community acquired infections and limit those with sepsis after nosocomial infections, post-surgical sepsis, and transfer patients who may have missed the opportunity for early vitamin C therapy. These factors have been identified as potential confounders of treatment effectiveness, as nosocomial infections may be more likely to be drug resistant and more difficult to treat [28]. Patients with admission glomerular filtration rates (GFR) less than 35 ml/min (measured by the Modification of Diet in Renal Disease (MDRD) formula [29]), an initial creatinine greater than 4.0 mg/dL (KDIGO Stage 3 disease), or a diagnosis of end stage kidney disease or prior kidney transplant were excluded from the study. This was done to allow for the temporal assessment of development of AKI and to prevent the inclusion of individuals with poor kidney function.

The following data were collected from the EHR: patient demographics; past medical history; visit characteristics (i.e., pre-identified medications administered, laboratory results, order sets ordered); primary and secondary diagnoses; hospital and ICU LOS; vasoactive drug use; and in-hospital mortality. Vasoactive drug use was defined as having received Dobutamine, Dopamine, Epinephrine, Norepinephrine, Phenylephrine, or Vasopressin. AKI was defined using ICD-10 code discharge diagnoses. Doses of IV vitamin C received were captured to categorize patients into two groups; comparison group (No or < 1.5 g of IV vitamin C), and treatment group (≥1.5 g of IV vitamin C within 12 h of hospital admission). Individuals who received ≥1.5 g of IV vitamin C more than 12 h after hospital admission were excluded from the study as research suggests that vitamin C treatment after the first 12 h may not be as efficacious [30]. Similarly, patients who received a couple of doses after 12 h and remaining within the time frame were also excluded to obtain a uniform comparable study sample. Of note, two patients in the comparison group received low doses of IV vitamin C of 0.5 g or less (Fig. 1).

### Statistical analysis plan

To assess bivariate associations, categorical data were compared using chi-square or Fisher’s exact test when appropriate, and continuous data were analyzed using independent-samples t-tests. Preliminary analyses revealed statistically significant demographic differences between individuals in the treatment versus comparison groups (Table 1). Considering the retrospective nature of the study where the treatment was not randomly assigned, we used propensity score matching (PSM) to balance the covariate distribution between groups. Further, as our treated and untreated groups are not directly comparable because they may systematically differ at baseline, propensity score matching plays an important role in balancing the study groups to make them comparable. The variables that were matched and selected for various subgroups are: insurance, race, biological sex, history of diabetes, history of hypertension, vasoactive drug use, and patient age. Vasoactive drug use was included as a marker of illness severity. The greedy method for matching was performed using a 3:1 ratio (comparison: treatment) to best match the groups’ sample size of the full study population. Utilizing the 3:1 ratio for matching the entire study population of sepsis patients meeting inclusion critiera (1390) was able to be used in the final analysis.

**Table 1:** Bivariate Analyses between IV Vitamin C Group and Comparison Group

	All Patients (*n* = 1390)		
Demographic Characteristic	IV vitamin C (*n* = 212)	Comparison (*n* = 1178)	p
Continuous	μ ± SE	μ ± SE	
Age (years)	62 ± 1.15	59 ± 0.51	0.02
Length of Stay (hours)	302 ± 18	319 ± 13	0.44
Length of Stay in ICU (hours)	119 ± 10	76 ± 6	< 0.001
Categorical	n (%)	n (%)	p
Insurance Status			
Government	182 (86)	893 (76)	0.001
Other	30 (14)	285 (24)	
Race			
Black	118 (56)	611 (52)	0.57
White	84 (39)	500 (42)	
Other/Unknown	10 (5)	67 (6)	
Biological Sex			
Female	100 (47)	542 (46)	0.76
Male	112 (53)	636 (54)	
Hypertension			
Yes	71 (33)	454 (39)	0.16
No	141 (67)	724 (61)	
Diabetes			
Yes	17 (8)	112 (10)	0.49
No	195 (92)	1066 (90)	
Vasoactive Drug Use*			
Yes	36 (17)	193 (16)	0.83
No	176 (83)	985 (84)	
AKI			
Yes	145 (68)	550 (47)	< 0.001
No	67 (32)	628 (53)	
Death			
Yes	30 (14)	89 (8)	0.002
No	182 (86)	1089 (92)	

*AKI* Acute Kidney Injury

*Vasoactive drug use was defined as having received at least one of the following: Dobutamine, Dopamine, Epinephrine, Norepinephrine, Phenylephrine, or Vasopressin

Using the matched sample, preliminary bivariate associations with primary outcome variables (i.e., AKI, mortality) were conducted and statistically significant and clinically meaningful variables were entered into two multiple logistic regressions. As the majority of patients (96%) who received high dose IV vitamin C were admitted to the ICU, subsequent analyses were conducted on the sub-sample of ICU patients. Similar to the full population, PSM using the aforementioned variables was performed to balance between treatment and comparison groups who were seen in the ICU, with matching performed using a 2:1 ratio (comparison: treatment) to match the ICU groups’ sample sizes. Preliminary bivariate analyses and multiple logistic regressions were then conducted on the ICU sample predicting AKI and mortality. A *p*-value of less than 0.05 and 95% confidence intervals (CI) not including one were considered significant. All analyses were performed using SAS 9.4 software.

## Results

### Sample demographics and Descriptives

Of the 26,346 patient admissions between 1 August 2016 and 31 December 2018, 16,159 were admitted via the ED. Of the ED admissions, 1390 were identified as having a diagnosis of sepsis alone, based on 42 ICD-10 codes and were included for analysis. Overall, diagnosis of AKI and in-hospital mortality rates were 50 and 9%, respectively. Patients were primarily male (54%), identified as black (52%) or white (42%), and had an average age of 59 years. The majority of patients had government insurance (77%), 38 and 9% of patients had a diagnosis of hypertension and diabetes, respectively, and 16% of patients received vasoactive drugs during their visit (see Table 2). Of the 1390 patients, 212 (15%) were identified as part of the IV vitamin C treatment group and 1178 (85%) were considered part of the comparison group. Within the treatment group, patients received high dose IV vitamin C, on average, 4.58 h after admission.

**Table 2:** Overall Descriptive Statistics (*n* = 1390)

Continuous Variables	μ ± SE
Age (years)	59 ± 0.46
Length of Stay (hours) Length of Stay in ICU (hours)	316 ± 11 82 ± 5
Categorical Variables	n (%)
Insurance Status	
Government	1075(77)
Other	315(23)
Race	
Black	729(52)
White	584(42)
Other/Unknown	77(6)
Biological Sex	
Female	642(46)
Male	748(54)
Hypertension	
Yes	525(38)
No	865(62)
Diabetes	
Yes	129(9)
No	1261(91)
Vasoactive Drug Use*	
Yes	229 (16)
No	1161 (84)
IV vitamin C Use	
Yes	212(15)
No	1178(85)
AKI	
Yes	695(50)
No	695(50)
Death	
Yes	119(9)
No	1271(91)

*AKI* Acute Kidney Injury

*Vasoactive drug use was defined as having received at least one of the following: Dobutamine, Dopamine, Epinephrine, Norepinephrine, Phenylephrine, or Vasopressin

Bivariate analyses revealed that individuals in the IV vitamin C group were slightly older (61 years versus 58 years, *p* = 0.02), had longer ICU LOS (119 h versus 76 h, *p* < 0.001), were less likely to have a history of hypertension (33% versus 39%, *p* = 0.02), more likely to have AKI (68% versus 47%, *p* < 0.001), and more likely to have died in the hospital (14% versus 8%, *p* = 0.002) compared to those in the comparison group (see Table 1). Given the differences between treatment and comparison groups, PSM was performed to balance covariates. The 3:1 matching reduced the full sample to *N* = 848 and the ICU group 2:1 matching reduced the ICU sample to *N* = 534. After PSM, preliminary analyses revealed that the treatment group was more likely to develop AKI (68% versus 49%, *p* < 0.001) and more likely to have died in the hospital (14.15% versus 8.02%, *p* = 0.01). Results of the multivariate logistic regressions are described below.

#### Acute kidney injury

A multivariate logistic regression determining adjusted odds ratio (AOR) revealed that age (AOR: 1.03 95%CI: 1.02–1.04), ICU LOS (AOR: 1.003 95%CI: 1.001–1.004), race (i.e., black compared to white; AOR: 1.59 95%CI: 1.12–2.16), sex (i.e., female compared to male; AOR: 0.70 95%CI: 0.52–0.93), and IV vitamin C (AOR: 2.07 95%CI: 1.46–2.93) were associated with an increased likelihood of AKI. Other covariates including insurance, hypertension, diabetes, and vasoactive drug use were not significant. See Table 3a.

**Table 3:** Multiple Logistic Regression Models Predicting Acute Kidney Injury and Mortality in the Total Population Performed after Propensity Score Matching (3:1) (*n* = 848)

a. Acute Kidney Injury			b. Mortality	
Variable	Adjusted-OR (95% CI)	***p***-value	Adjusted-OR (95% CI)	***p***-value
Age (years)	1.03 (1.02, 1.04)	< 0.001	1.03 (1.01, 1.05)	0.007
Length of ICU Stay (hours)	1.003 (1.001, 1.004)	< 0.001	1.002 (1.001, 1.003)	< 0.001
Insurance (ref = Other)	0.78 (0.51, 1.21)	0.27	1.03 (0.44, 2.39)	0.95
Race (ref = White)				
Black	1.59 (1.18, 2.16)	0.04	1.31 (0.78,	0.64
Other/Unknown	1.14 (0.58, 2.24)	0.76	2.20) 2.28 (0.83, 6.29)	0.16
Biological Sex (ref = Male)	0.70 (0.52, 0.93)	0.01	0.80 (0.48, 1.32)	0.38
Hypertension (ref = No)	0.78 (0.57, 1.07)	0.12	1.30 (0.77, 2.20)	0.33
Diabetes (ref = No)	0.85 (0.50, 1.45)	0.55	0.87 (0.33, 2.33)	0.78
Vasoactive Drug Use* (ref = No)	1.54 (0.98, 2.40)	0.06	1.46 (0.81, 2.63)	0.21
IV vitamin C (ref = No)	2.07 (1.46, 2.93)	< 0.001	1.67 (1.003, 2.78)	0.04
AKI			3.21 (1.70, 6.03)	< 0.001

*AKI* Acute Kidney Injury

*Vasoactive drug use was defined as having received at least one of the following: Dobutamine, Dopamine, Epinephrine, Norepinephrine, Phenylephrine, or Vasopressin

#### Mortality

A multivariate logistic regression predicting mortality revealed that age (AOR: 1.03 95%CI: 1.007–1.05), ICU LOS (AOR: 1.002 95% CI: 1.001–1.003), diagnosis of AKI (AOR: 3.21 95%CI: 1.70–6.03), and IV vitamin C (AOR: 1.67 95% CI: 1.003–2.78) were associated with in-hospital mortality. Insurance, race, hypertension, diabetes, and vasoactive drug use were not significant. See Table 3b.

### Sub-analysis on ICU patients only (*n* = 534)

#### Acute kidney injury

A multivariate logistic regression performed on the sub-set of individuals who spent time in the ICU found that age (AOR: 1.02 95% CI: 1.01–1.04), ICU LOS (AOR: 1.003 95% CI: 1.001–1.004), and IV vitamin C (AOR: 1.61 95% CI: 1.09–2.39) were significant predictors of AKI. All other covariates were not significant. See Table 4a.

**Table 4:** Multiple Logistic Regression Models Predicting Mortality and Acute Kidney Injury in the ICU Population Performed after Propensity Score Matching (2:1) (*n* = 534)

a. Acute Kidney Injury			b. Mortality	
Variable	Adjusted-OR (95% CI)	***p***-value	Adjusted-OR (95% CI)	***p***-value
Age (years)	1.02 (1.01, 1.04)	< 0.001	1.03 (1.007, 1.04)	0.01
Length of ICU Stay (hours)	1.003 (1.001, 1.004)	<.001	1.001 (1.000, 1.002)	0.05
Insurance (ref = Other)	0.93 (0.56, 1.54)	0.78	1.51 (0.68, 3.33)	0.31
Race (ref = White)				
Black	1.47 (0.99, 2.19)	0.04	1.56 (0.94, 2.60)	0.85
Other/Unknown	0.71 (0.28, 1.78)	0.24	2.77 (0.88, 8.73)	0.16
Biological Sex (ref = Male)	0.77 (0.52, 1.12)	0.17	0.67 (0.41, 1.11)	0.12
Hypertension (ref = No)	0.72 (0.49, 1.08)	0.11	1.18 (0.70, 1.96)	0.54
Diabetes (ref = No)	1.39 (0.68, 2.83)	0.37	0.47 (0.16, 1.38)	0.17
Vasoactive Drug Use* (ref = No)	1.37 (0.84, 2.24)	0.20	0.90 (0.50, 1.63)	0.72
IV vitamin C (ref = No)	1.61 (1.09, 2.39)	0.02	0.79 (0.48, 1.31)	0.36
AKI			2.01 (1.12, 3.59)	0.02

*AKI* Acute Kidney Injury

*Vasoactive drug use was defined as having received at least one of the following: Dobutamine, Dopamine, Epinephrine, Norepinephrine, Phenylephrine, or Vasopressin

#### Mortality

A multivariate logistic regression revealed that age (AOR: 1.03 95% CI: 1.01–1.04) and AKI (AOR: 2.01 95% CI: 1.12–3.59) were significant predictors of in-hospital mortality. All other variables in the regression analysis, including IV vitamin C, were not significant. See Table 4b.

## Discussion

This retrospective study of patients hospitalized for sepsis revealed an association between IV vitamin C usage and subsequent AKI and in-hospital mortality. When examining the association of IV vitamin C therapy and AKI, the odds of experiencing AKI for individuals treated with IV vitamin C were 107% higher than the odds for individuals not treated with IV vitamin C, after controlling for a number of demographic and clinical variables. Findings also revealed that risk of AKI was increased for those who were male and older, which is supported in previous research [31, 32]. Findings are consistent with results from a similar study of patients admitted via the ED for sepsis, which found that individuals over 65 years were at significantly higher risk of AKI and subsequent in-hospital death [33]. Other research has also highlighted the relationships between increased LOS and older age, and the occurrence of AKI and mortality [34, 35]. Other notable factors in the current study associated with increased risk of AKI were longer ICU LOS and identifying as black as compared to white, both of which are risk factors supported by previous research [36–38]. The association between IV vitamin C and AKI is not unique to this study; a study by Litwak et al. observed a 12% increase in AKI in patients that received IV vitamin C but this did not reach statistical significance, likely due to small sample size [22]. Additionally, this relationship between IV vitamin C and AKI has been identified in several case reports [23–25]. The results from the current study differ from a large clinical trial (VICTAS) and a smaller retrospective study that found a beneficial relationship between IV vitamin C and AKI. The disparate results may also be explained by sample size and related power; the VICTAS study was ended earlier than planned and the retrospective study had a small sample size of only 47 patients [15, 26].

When examining in-hospital mortality, results revealed that being older, having AKI, longer ICU LOS, and receiving IV vitamin C were associated with increased risk of mortality. Specifically, the odds of mortality were 67% higher for the IV vitamin C group compared to those in the comparison group. The association between high dose vitamin C and death was unexpected and not easily explained. Another variable found to be associated with an increased risk of death, which may lend insight into the relationship between vitamin C and mortality, was the diagnosis of AKI. Previous research indicates kidney failure increases the risk of death six to eight times in septic patients [39]. However, when examining the data in the current study, an increased risk of death was evident irrespective of AKI. Among patients who did not have AKI and did not receive IV vitamin C, 3.03% died compared to 7.46% of those who did not have AKI but received IV vitamin C. This suggests there may be a direct effect of high dose vitamin C that may exacerbate the risk of mortality. The current study lacked the data that would allow for a more comprehensive examination between high doses of IV vitamin C and potential increased toxicity outside of the kidney, however, future research should explore the relationship between vitamin C and mortality, irrespective of AKI.

The current finding that IV vitamin C is associated with increased risk of death is inconsistent with another large retrospective study which found IV vitamin C to be associated with reduced mortality among patients with sepsis [21]. One reason for the disparate results may be differences in treatment dose. Specifically, vitamin C in the Byerly et al. study was not limited to high dose and was not defined by a threshold defining the treatment group, whereas the current study examines what is considered *high dose* IV vitamin C (≥1.5 g), which may account for outcome differences. One study that examined higher doses (200 mg/kg/day) of vitamin C showed evidence of increasing rates of mortality, with higher mortality (50.6%) among a high dose vitamin C group compared to patients who received lower doses of vitamin C (50 mg/kg/day) (38%), though both treatment groups had lower mortality than the placebo group (63%) [14]. Notably, this study was limited by a small sample size and did not reach statistical significance. Additional research examining other clinical markers including SOFA scores in patients receiving high dose IV vitamin C over long periods of time is needed.

Sub-analyses of the ICU sample revealed that age, longer ICU LOS, and receiving IV vitamin C remained predictors of AKI, while identifying as black and being male were no longer significant predictors. Older age, and ICU LOS are both known risk factors supported by previous research [36–38]. A second sub-analysis revealed that age and AKI remained significant predictors of in-hospital mortality, however high dose IV vitamin C use was no longer a statistically significant predictor of mortality once the sample was limited to ICU patients only.

The current study observed a moderate relationship between IV vitamin C and mortality in septic patients, which might suggest potential nephrotoxicity of vitamin C therapy. The association between high dose vitamin C and AKI due to oxalate deposition in renal tubules is well established [40]. Since early reports in 1985, there are multiple cases of AKI resulting from high dose vitamin C, administered parenterally or orally [23, 41–44]. Ascorbic acid is metabolized intracellularly and converted to oxalic acid [45]; the oxalic acid is then filtered in the kidney where it precipitates into crystals of calcium oxalate, potentially causing obstruction and tubular injury. Previous research has shown that vitamin C doses as low as 480–960 mg/D, taken orally over several months, have resulted in oxalate deposition kidney failure that requires temporary dialysis [46]. Dietary supplementation of 2 g per day can increase oxalate excretion by 21.8% [47]. Oxalic acid and oxalate toxicity have been shown to occur in myocardial tissue of patients with hereditary hyperoxaluria [48]*.* Others have suggested that similar myocardial tissue damage can occur in secondary forms of hyperoxalosis [49, 50]. Acutely elevated oxalate levels in the serum could cause myocardial dysfunction, leading to increased risk of mortality regardless of AKI.

### Limitations

This study was limited in the retrospective nature of the design, which precludes causal-effect conclusions and prevents patient follow-up. Relatedly, the EHR data utilized did not include time stamps for diagnoses and treatment, thus temporal order is unknown. However, strict inclusion and exclusion criteria were applied in an effort to reduce selection bias and focus on community-acquired sepsis. Further, as vasoactive drug use and ICU admission were the only available surrogates for illness severity, these factors were considered in the PSM and sample stratification, respectively. Although the current study was limited to one hospital, the large sample size provided adequate power to detect statistically significant associations between patient-level demographic and clinical variables, and specifically, vitamin C usage, AKI, and mortality. Additionally, existing literature has commonly reported SOFA scores, which were unable to be utilized in the current study due to inconsistencies in collection. Many studies have used SOFA scores as a representation of mortality risk, whereas in the current study, we were able to directly examine in-hospital mortality as a primary outcome. Additionally, the usage of serum lactic acid and procalcitonin levels were reviewed as a potential measure of disease severity but could not be included in analyses due to data missing not at random. In other words, these laboratory tests were not performed equally across all groups of patients. Finally, the current study did not assess for other nephrotoxic drugs that could be related to AKI, such as antimicrobials and radiologic contrast dye. Future research should include these as well as other factors to assess the unique relationship between IV vitamin C and AKI.

## Conclusion

Vitamin C is required for many biological functions including tissue repair, proper immune function, and healing. In sepsis patients, vitamin C levels are depleted thus IV vitamin C therapy is proposed as a mechanism of sepsis treatment. This retrospective review of patients with sepsis revealed though that early use of high dose IV vitamin C may *increase* the likelihood of AKI and does *not* serve as a protective factor against mortality. The association of vitamin C with AKI, along with the association of AKI and mortality in patients admitted to the ICU, suggests that high doses of vitamin C may not be beneficial but toxic. This finding, coupled with results from multiple randomized controlled prospective studies of IV vitamin C with mixed results, suggests that wide-spread use of high dose IV vitamin C requires further assessment. Research examining whether there are significant differences in IV vitamin C therapy toxicity among sub-group populations is necessary to further identify appropriate usage of IV Vitamin C therapy. These investigations should also include the different sources of sepsis related to hospital admissions, including those admitted through other routes than the emergency department. Additional research utilizing existing hospital data from across the nation and worldwide are needed to better understand the relationship between high dose IV vitamin C therapy and subsequent outcomes as well as determine the toxic/effective dose.

## Data Availability

The data that support the findings of this study were obtained from Sentara Healthcare, and restrictions apply to the availability of these data, which were used under the Sentara Healthcare and EVMS-Sentara Healthcare Analytics and Delivery Science Institute agreement for this study. Sentara Healthcare will consider sharing this data upon request. The data are not publicly available because they contain information that could compromise the privacy of the research participants. Requests to review the data can be made to Dr. Sunita Dodani MD PhD, Director, EVMS-Sentara Healthcare Analytics and Delivery Science Institute, Eastern Virginia Medical School, Norfolk, Virginia. Email: dodanis@evms.edu.

## Abbreviations

AKI: Acute Kidney Injury
AOR: Adjusted Odds Ratio
CI: Confidence Interval
ED: Emergency Department
EVMS: Eastern Virginia Medical School
GFR: Glomerular Filtration Rate
HER: Electronic Health Record
ICD: International Classification of Diseases
ICU: Intensive Care Unit
IV: `Intra Venous
KDIGO: Kidney Disease Improvement Global Outcome
LOS: in hospital Length of Stay
MDRD: Modification of Diet in Renal Diseases
OR: Odds Ratio
PSM: Propensity Score Matching
SAS: Statistical Analysis System
SOFA: Sequential Organ Failure Score
VICTAS: Vitamin C, Thiamine, and Steroids in Sepsis
WI: Wisconsin
